# LONELINESS AMONG SEXUAL AND GENDER DIVERSE PEOPLE IN AUSTRALIA: AN AGE-BASED ANALYSIS

**DOI:** 10.1093/geroni/igae098.1090

**Published:** 2024-12-31

**Authors:** Mark Hughes, Karen Fredriksen-Goldsen

**Affiliations:** Southern Cross University, Bilinga, Queensland, Australia; University of Washington, Seattle, Washington, United States

## Abstract

Despite recent equality reforms, there remain significant mental health disparities between sexual and gender diverse (SGD) Australians and the general population. Multiple studies have reported higher levels of loneliness among SGD people, particularly among transgender and nonbinary people. One factor related to loneliness that is much debated is age with some studies suggesting older people are more at risk and others finding contradictory evidence, including among SGD people. To examine age-based loneliness trends among different groups of SGD people, we analyzed Australian data (n = 284) from the Global Pride Study to examine how key individual and social variables differ by age and the extent they predict loneliness (measured by the 3-item Loneliness Scale). Through regression analysis, we identified that gender and sexual orientation did not appear as predictors of loneliness. However, being younger did emerge as a predictor of a higher level of loneliness, as did lower levels of social support and resilience. Past experiences of SGD discrimination and victimization and a greater number of functional limitations also predicted higher levels of loneliness. Given recent reforms, one might expect younger SGD people to be faring better than older people on social and wellbeing indicators such as loneliness. However, this study provides some support to prior research indicating many older SGD people have developed considerable resilience across the lifespan and strengthened support systems later in life in a way that may ameliorate loneliness.

